# Effectiveness of Activated Sodium Hypochlorite Irrigation by Shock Wave-Enhanced Emission Photoacoustic Streaming, Sonic and Ultrasonic Devices in Removing *Enterococcus faecalis* Biofilm From Root Canal System

**DOI:** 10.3390/jcm13206278

**Published:** 2024-10-21

**Authors:** Hadi Assadian, Sadaf Fathollahi, Maryam Pourhajibagher, Luca Solimei, Stefano Benedicenti, Nasim Chiniforush

**Affiliations:** 1 Department of Endodontics, School of Dentistry, Tehran University of Medical Sciences, Tehran 14399-55991, Iran; hd.asdn@scident.ir; 2 Private Practice, Tehran 19479-53893, Iran; sadaffathollahi@yahoo.com; 3 Dental Research Center, Dentistry Research Institute, Tehran University of Medical Sciences, Tehran 14155-5583, Iran; mphb65@yahoo.com; 4 Department of Surgical Sciences and Integrated Diagnostics, University of Genoa, Viale Benedetto XV, 6, 16132 Genoa, Italy; lucasolimei@hotmail.it (L.S.);

**Keywords:** biofilms, dental pulp cavity, disinfection, lasers, root canal therapy

## Abstract

**Aim:** To compare shock wave-enhanced emission photoacoustic streaming (SWEEPS) with sonic- and ultrasonically activated irrigation systems in removing *Enterococcus faecalis* biofilm from the root canal system. **Methodology:** Fifty human single-canalled mandibular premolars were included in the study. After access cavity preparation, the root canals were prepared to a standardized size and taper. Then, the entire root surface was covered with two layers of resin, and the root apices were sealed before sterilization. All root canals were inoculated with *E. faecalis* biofilm, and the samples were incubated aerobically for 2 weeks at 37 °C. Biofilm formation was confirmed by scanning electron microscopy. All samples were randomly divided into five groups (*n* = 10 each) based on their irrigation activation method as A (no treatment or negative control), B (no irrigation or positive control), C (sonically activated irrigation (SAI)), D (ultrasonically activated irrigation (UAI)), and E (needle irrigation activated by an Er: YAG laser device using a SWEEPS quartz tip (SWEEPS)). Then, dentine chips were retrieved, vortexed, and diluted for colony-forming unit counts. Data were analysed using analysis of variance and post-hoc Tukey tests (α = 5%). **Results:** All methods could significantly reduce *E. faecalis* biofilm compared with control so that the UAI, SWEEPS, and SAI groups indicated a 23.54%, 14.89%, and 7.81% biofilm reduction, respectively. UAI demonstrated a significantly more effective reduction of *E. faecalis* biofilm than SAI (*p* = 0.004). **Conclusions:** All irrigation activation methods significantly reduced *E. faecalis* biofilm, with ultrasonic use being the most effective.

## 1. Introduction

The elimination of microorganisms and their byproducts through the chemomechanical preparation of the root canal system is considered one of the most important aims of root canal treatment [1]. It has been demonstrated that persisting bacteria following endodontic treatment can negatively influence the treatment outcome [2]. Therefore, optimizing chemomechanical disinfection through various chemical solutions, regimens, and delivery systems is proposed in line with variable instrumentation techniques [3]. Studies have demonstrated that microorganisms are able to reside in the root canal system, form a biofilm, and invade dentinal tubules, thereby evading chemomechanical debridement [4]. Therefore, the disruption of the created biofilm through debridement procedures in infected root canal systems is a crucial step in successful treatment [5]. Endodontic infections are polymicrobial in nature and are mainly caused by anaerobes and some facultative bacteria [6]. Numerous studies have focused on *Enterococcus faecalis* as a facultative microorganism to be the culprit in persisting, as well as primary endodontic infections, due mainly to its capability to form a biofilm and reside in nutrition-deprived environments [7,8,9]. It has been shown that the passive use of irrigating solution only does not meet the minimum requirements of the root canal disinfection; hence, several activation procedures have been proposed to enhance the efficiency of debridement including sonic- (SAI) and ultrasonic-activated irrigation (UAI). In SAI, the root canal space is pooled with the irrigant, and a specified metal file or polymer tip is inserted into the solution and is activated at a frequency of less than 20,000 Hz. Endo Activator (EA; Dentsply, Tulsa Dental Specialties, Tulsa, OK, USA) is a cordless and battery-operated device that can be used for SAI and uses three sizes of polymer tips (yellow 15/02, red 25/04, and blue 35/04) at three speeds i.e., 2000, 6000, and 10,000 cpm. Arslan et al. [10] have demonstrated that SAI using EA was superior to passive needle irrigation in removing double and triple antibiotic pastes from root canals in vitro. Also, Balić et al. [11] showed that EA could effectively remove *E. faecalis* biofilm in preclinical conditions. The use of SAI and UAI is supported based on the formation of acoustic microstreaming and hydrodynamic cavitation as their underlying activation mechanism. In UAI, acoustic energy is delivered at the frequency of >20,000 Hz to a non-cutting file within the irrigating solution. Sabins et al. demonstrated the superiority of UAI over conventional and SAI in cleaning efficacy [12]. Also, in a study by Rödig et al. [13], a more effective elimination of debris from simulated root canal irregularities was observed in ultrasonic irrigation in comparison with the Vibringe System and syringe irrigation.

In recent years, laser-activated irrigation (LAI) has been recommended by some authors to improve the efficiency of endodontic debridement [3,10,14]. In this context, the use of erbium lasers has been found useful due to their higher water absorption coefficient. It has been demonstrated that strong cavitations and shockwaves can be produced while erbium lasers are being used at lower energies during irrigation activation [15]. As a result, the removal of dentinal debris and the smear layer can be observed due to the rapid movement of the intra-canal irrigant and the high shear stress on the root canal wall [16]. It has been demonstrated that the use of photon-initiated photoacoustic streaming (PIPS) in endodontic irrigation resulted in photoacoustic pressure waves within the root canal irrigant without any thermal damage to dentinal walls. While using PIPS for irrigant activation, the conical and stripped fiber tip is inserted within the access cavity and the irrigant is streamed by minute ruptures of low laser energy beams. More recently, a shock wave-enhanced emission photoacoustic streaming (SWEEPS) technique was introduced to enhance disinfection in the confined environment of the root canal space and has many common features with extracorporeal shock wave lithotripsy (ESWL) to treat nephrolithiasis. In this technique, an erbium-doped yttrium aluminum garnet (Er:YAG) laser with extremely short pulses (50 μs) is used. A second laser pulse is delivered into the irrigant immediately after the collapse of the first cavitation bubble to create a second bubble which, in turn, accelerates the collapse of the first cavitation bubble. The violent collapse of the created cavitation bubbles can result in the emission of shockwaves during the laser-activated irrigation of the root canal space. The high absorption of the short laser pulse in the irrigant solution results in the prompt creation of vapor bubbles due to a sudden temperature rise near the fiber tip [17]. Several studies have demonstrated the superior performance of PIPS/SWEEPS over conventional irrigation such as SAI and UAI. For example, Dönmez Ozkan et al. [18] showed PIPS method to be more effective in removing biomolecular film than passive ultrasonic irrigation (PUI), SAI (EA), and conventional irrigation. In addition, Arslan et al. [10] demonstrated that PUI led to the greater but incomplete removal of calcium hydroxide, whereas the PIPS method led to the complete removal of this material from artificial grooves in the straight root canals.

Although studies comparing the efficacy of different activated irrigation systems on removing *E. faecalis* biofilm are numerous, to the best of the authors’ knowledge, no study has compared SWEEPS with activated irrigation systems (SAI and UAI).

## 2. Materials and Methods

The investigation was conducted according to the Ethical Approval Code No. 30224856 issued by Ethical Community of Biomedical Research.

### 2.1. Sample Selection and Preparation

A total of 50 mandibular premolars which were extracted for periodontal reasons were selected. The external root surfaces were cleaned with a curette to remove calculus and remaining periodontal tissues. The teeth were disinfected using 3% sodium hypochlorite solution (NaOCl) at room temperature for 24 h and were stored in distilled water until use [19]. The presence of a single round canal was confirmed by radiographs taken in both buccolingual and mesiodistal directions. The teeth were confirmed to have no more than one canal, previous root canal treatment, internal or external resorption, calcification, immature root apex, cracks/fractures, or decay at the root surface or curved canals. The coronal parts of the teeth were preserved in order to have reservoirs for the irrigant. After the preparation of the access cavity, an ISO size #10 K-file (Dentsply Maillefer, Ballaigues, Switzerland) was inserted into the canal, and, as soon as the file appeared from the apical part of the main foramen, the working length was calculated by reducing this length by 1 mm. The canals were prepared up to size F3 ProTaper Universal (Dentsply/Tulsa Dental Specialties, Tulsa, OK, USA). The canals were irrigated by 2 mL of 5% NaOCl between the exchange of each instrument. The final irrigation was performed using 5 mL of 5% NaOCl solution followed by 5 mL of 17% EDTA solution, each for 1 min. The canals were then rinsed with 5 mL of distilled water and then dried with paper points (Dentsply Maillefer, Ballaigues, Switzerland). Then, two layers of dentine bonding material (Gaenial Bond, GC, Tokyo, Japan) were applied to the entire root surface. The root apices were then sealed with glass ionomer cement (Kavitan_Plus; Pentron, SpofaDental, Jičín, Czech Republic) in order to create a closed-end and steam-lock effect according to Tay et al. [20]. The samples were packed and sterilized by autoclave at 121 °C and 15 MPa for 30 min. All procedures were meticulously conducted under aseptic conditions to maintain the sterility and integrity of the samples.

### 2.2. Bacterial Biofilm Inoculation

A pure culture of *E. faecalis* ATCC 29,212 (Iranian Biological Resource Center, Tehran, Iran) was utilized to establish a biofilm within root canals. The bacteria were grown in brain heart infusion (BHI) broth (Merck, Darmstadt, Germany) incubated at 37 °C for 24 h. The inoculum density was adjusted until the turbidity matched the 0.5 McFarland standard, equating to approximately 1.5 × 10^8^ colony-forming units per milliliter (CFUs/mL). The prepared *E. faecalis* suspension was introduced into each root canal using a 3.0 mL syringe fitted with a 27-gauge needle. Each tooth was individually placed in a sterile microtube and maintained at 37 °C under aerobic conditions for 2 weeks. The bacterial suspension was renewed every two days to promote the development of a mature biofilm.

### 2.3. Antibacterial Regimen

The samples were randomly divided into five groups (*n* = 10 each) and classified according to the irrigation activation method. In all groups, the total irrigation time was 90 s, and the total irrigant volume consisted of 3 mL of 5.25% NaOCl solution. The total activation time for all groups was 60 s. Irrigant activation in the experimental groups was carried out as follows:

Group A (negative control group): No treatment was carried out after sterilization.

Group B (positive control group): No irrigation was done after bacterial contamination.

Group C: The root canal was irrigated for 10 s with a 27-gauge Endo-Eze ^®^ (Ultradent Inc., South Jordan, UT, USA) needle with a notched tip, using 1 mL of 5.25% NaOCl for three consecutive times. Activation was performed by the EndoActivator (Dentsply/Tulsa Dental Specialties, Tulsa, OK, USA) at a speed of 10,000 cycles/min using the red tip (size#25/0.04) for 20 s. The tip of the device was placed 2 mm short of the working length, and the cycles were repeated 3 times. The root canals were then dried with sterile paper points.

Group D: After rinsing the canal with 1 mL of 5.25% NaOCl by the same needle as described, the ultrasonic tip (size #15/0.02) was placed inside the root canal 2 mm short of the working length for 10 s. The tip was activated for 20 s at a frequency of 28–32 kHz. This process was repeated 3 times. The root canals were then dried with sterile paper points.

Group E: The root canals were irrigated with 0.5 mL of 5.25% NaOCl solution for 10 s, and then activation was performed by an Er: YAG laser device (Light walker, Fotona, Slovenia) with a wavelength of 2940 nm, using a SWEEPS 600 μm quartz tip at a power of 0.3 W, a frequency of 15 Hz, and 20 mJ energy per pulse in Auto SWEEPS mode. The water and air sprays of the device were switched off. First, the root canal was irrigated with 0.5 mL of NaOCl solution for 10 s. The optical fiber of the device was inserted into the access cavity, and then the device was activated for 20 s. Whenever the irrigant was reduced in the access cavity, an additional 0.5 mL of 5.25% NaOCl solution was added to fill the access cavity, and the device remained active during this process. The whole process was repeated 3 times, and the root canals were subsequently dried with sterile paper points.

### 2.4. Microbiological Evaluation

The dentine chips, weighing approximately 0.01 ± 0.002 g, were collected from the root canal walls using size F3 ProTaper Universal drills (Dentsply/Tulsa Dental Specialties, Tulsa, OK, USA) to investigate the antimicrobial effects of various activated irrigation systems. To ensure uniformity and standardization across all samples, the specimens were meticulously harvested from the coronal third of each root canal wall, precisely 2 mm apical to the cemento-enamel junction (CEJ) of each tooth root.

These chips were separately transferred into sterile microtubes containing 1 mL of sterile BHI broth medium, followed by vortexing for 20 s. Subsequently, 10 μL from each microtube was serially diluted, and 10 μL of each dilution was cultured on BHI agar (Merck, Darmstadt, Germany) using the spread plate protocol by a sterile spreader. After incubating for 24 h at 37 °C, the CFUs/mL in each sample were counted based on the method by Miles et al. [21].

### 2.5. Scanning Electron Microscopic (SEM) Evaluation

**Figure 1:**
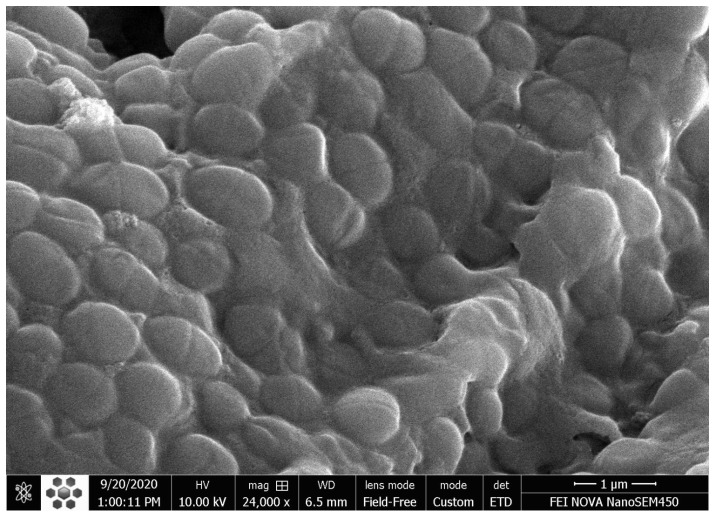
Scanning electron micrograph showing accumulation of *E. faecalis* colonies on the surface of the root canal wall.

SEM analysis was performed for two samples that were randomly selected from the positive control group to confirm biofilm formation [22]. The sample was dehydrated using incremental concentrations of ethanol and acetone prior to gold-palladium coating. The sample was then visualized by SEM device (5800LV, JEOL, Tokyo, Japan) under 50–24,000× magnification at 10.00 kV (Figure 1).

### 2.6. Statistical Analysis

The statistical analysis was performed using SPSS software (version 23.0, IBM Corp., Armonk, NY, USA). The Kolmogorov–Smirnov test was used to evaluate the normal distribution of CFUs in experimental and control groups. One-way analysis of variance (ANOVA) and relative variance were used to determine whether there were any statistically significant differences between the means of the experimental groups. The magnitude of the effect was also calculated using Cohen’s d.

## 3. Results

The CFU counts for *E. faecalis* showed a normal distribution according to the Kolmogorov–Smirnov test. (Table 1) All three experimental methods (SAI, UAI, and SWEEPS) were effective in removing *E. faecalis* biofilm according to the one-way ANOVA test. Furthermore, the magnitude of their effect was considered large according to Cohen’s test. According to the Tukey post hoc test, both the SWEEPS and UAI groups showed a significant reduction of the formation of *E. faecalis* biofilm in comparison to the positive control group (*p* = 0.007 and 0.001, respectively). UAI and SWEEPS showed a significant reduction of *E. faecalis* biofilm in comparison with the SAI group (*p* = 0.004 and 0.007, respectively) (Table 2).

The calculation of the relative mean changes indicated that UAI, SAI, and SWEEPS had decreases of 23.54%, 7.78%, and 2.6% in *E. faecalis* biofilm compared to the positive control group, respectively (Table 3).

## 4. Discussion

The removal of bacterial biofilm from the root canal system plays a pivotal role in the success of endodontic treatment. Siqueira et al. [23] have demonstrated that the persistence of bacteria within the untouched portions of the root canal system during chemomechanical preparation can negatively influence the outcome of endodontic treatment. This can occur mainly due to bacteria, their products, and necrotic tissue, which provide an environment that can lead to bacterial regrowth and consequent apical periodontitis [1,24,25]. It has been advised by some authors that the efficiency of endodontic disinfection can be enhanced by activated irrigation strategies such as LAI, UAI, and SAI [26,27,28]. However, the irrigation methods used in contemporary endodontics are not capable of complete biofilm removal according to the literature. *E. faecalis* is used as a typical microorganism for the evaluation of root canal system disinfection due to its established role in secondary endodontic infections [29,30]. Therefore, in this study, it was decided that we would evaluate the effect of SAI, UAI, and SWEEPS as contemporary activated irrigation strategies for the removal of *E. faecalis* biofilm.

**Figure 2:**
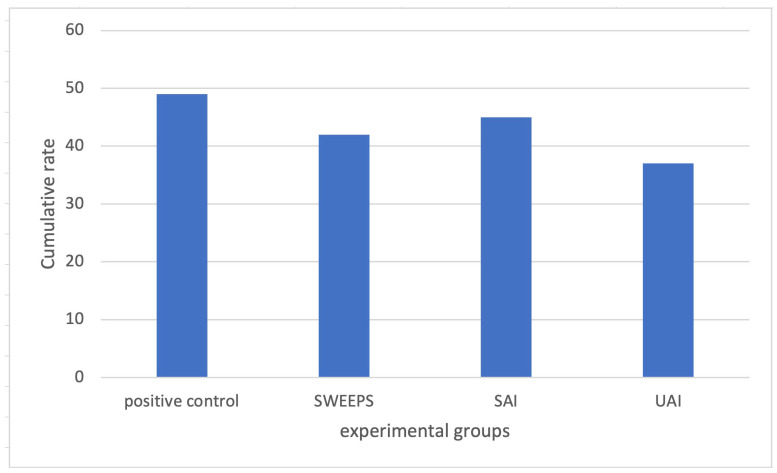
Bacterial culture in different activation methods compared to the positive control group.

In the current investigation, it was shown that UAI and SWEEPS methods could significantly reduce the biofilm of *E. faecalis* compared to the positive control group (Figure 2). This was in agreement with various studies, including De Meyer et al. [31], Race et al. [32], and Neelakantan et al. [33], in which the authors declared that activated irrigation strategies led to a significant reduction in bacterial biofilm compared to the control.

It was found in the present study that PUI was more effective than SAI and SWEEPS in the eradication of *E. faecalis* biofilm, while Arslan et al. [10] showed that PIPS was more effective than SAI and UAI techniques in removing organic debris in the apical canal area. Such variability can be attributable to differences in activation protocols, laser tips used, and the root canal areas to be examined.

In the current investigation, UAI showed significantly more effective biofilm removal than SAI and SWEEPS. This can be attributable to the increased depth of irrigant penetration in comparison with the other two methods. This was in part corroborated by Niavarzi et al. [34], who demonstrated that a greater penetration depth of the photosensitizer was observed in ultrasonically activated photodynamic therapy.

In the present study, the activation of sodium hypochlorite by SWEEPS showed a significant reduction in *E. faecalis* biofilm compared with the control. This finding was in agreement with Wang et al. [35], who found that *E. faecalis* biofilm showed a greater decrease by SWEEPS-activated sodium hypochlorite (20 mJ, 15 Hz, 0.3 W, 2.94 μm, water and air off) in comparison with the PIPS-activated group (20 mJ, 15 Hz, 0.3 W, 2.94 μm, water and air off) and passive sodium hypochlorite irrigation within the palatal and distal roots of maxillary molars.

Due to the very high absorption coefficient of the Er: YAG laser wavelength (λ = 2940 nm), all laser pulse energy is absorbed in the liquid layer to a thickness of approximately 1 μm. Therefore, the liquid heats up locally and immediately exceeds the boiling point, causing a vapor bubble to begin forming at the end, which then expands. When it reaches its maximum volume, it begins to disintegrate due to the pressure of the surrounding liquid. This phenomenon causes turbulent fluid movement throughout the entire volume of the root canal and significantly improves chemomechanical debridement [36]. Contrary to large fluid reservoirs, shock waves, which are waves that travel faster than sound, are not seen in limited space reservoirs such as the root canal. This is because, in narrow canals, the friction of the canal walls and the limited space available for rapid fluid displacement during bubble expansion and contraction significantly slows down the process [37]. SWEEPS mode involves delivering the next laser pulse to the liquid at an optimal time, when the initial bubble is in the final stage of disintegration. The growth of the second bubble exerts pressure on the collapsing primary bubble, accelerating its collapse and the breakdown of the secondary bubbles, resulting in the propagation of the primary waves as well as the secondary shock waves [38]. This was corroborated by Wang et al. [35], who showed that SWEEPS significantly reduced, if not eliminated, bacteria and the smear layer compared to PIPS.

Since the laser tip in the SWEEPS technique is located in the access cavity and is far from the apex, the attenuation of the photoacoustic effects may be the reason for its less effective biofilm removal compared to the SAI and UAI, which have a more intimate contact with the target location. In addition, the laser settings may not be strong enough to cause cavitation and streaming in the apical region.

A significant energy transfer to the irrigant can happen by using SWEEPS and UAI inside the root canal space. On the other hand, the energy created by using SAI is insufficient to create cavitation, as stated by Jiang et al. [39]. This can be attributed to the lower impact of SAI in *E. faecalis* biofilm removal compared to UAI and SWEEPS.

It cannot be overemphasized that the results of this ex vivo study should not be directly extrapolated to clinical settings. However, the use of multi-species biofilms in variable tooth types and different microbiological assessments could provide more insight into the antibacterial effectiveness of activated irrigation systems in endodontics.

## 5. Conclusions

All three irrigation activation methods (SAI, UAI, and SWEEPS) had a significant impact on bacterial biofilm elimination compared to the control. The UAI method was significantly more effective than both SAI and SWEEPS in removing *E. faecalis* biofilm.

## Figures and Tables

**Table 1:** Dispersion distribution of *E. faecalis* colony formation in different root canal irrigation activation systems (SAI, UAI, and SWEEPS) CFU/mL × 10^5^.

Groups (*n* = 10 Each)	Domain		Average	Standard Deviation	Significance Level Kolmogorov Smirnov
	Maximum	Minimum			
Negative control	0	0	0	0	0
Positive control	56.40	40.60	49.02	5.18	0.99
SWEEPS	50.50	36.30	41.72	4.99	0.66
SAI	52	39.10	45.19	4.78	0.75
UAI	43.20	31.40	37.48	3.62	0.98

**Table 2:** Comparative effects of different root canal irrigation activation systems (SAI, UAI, and SWEEPS) in removing *E. faecalis* biofilm (CFU × 10^5^).

Group	Comparison Group	Mean Difference ^1^	Standard Deviation	*p*	Confidence Interval 95%	
					Maximum	Minimum
SWEEPS	positive control	−7.30	2.09	0.007	−1.65	−12.94
	SAI	−3.47	2.09	0.36	2.17	−9.11
	UAI	4.24	2.09	0.19	9.88	−1.40
SAI	positive control	−3.83	2.09	0.27	1.81	−9.47
	UAI	7.71	2.09	0.004	13.35	2.06
UAI	positive control	−11.54	2.09	0.001	−5.89	−17.18

^1^ The column indicates mean CFU differences compared with positive control group; Negative values show less and positive ones more CFU values compared with positive control.

**Table 3:** Relative changes in different root canal irrigation activation systems (SAI, UAI, and SWEEPS) in removing *E. faecalis* biofilm compared to negative control group.

100 × $\frac{Group\;average-Control\;average}{Control\;average}$	**Relative Change Rate (%)**	**Group Comparison**
	−14.89	SWEEP with positive control
	−7.81	SAI with positive control
	−23.54	UAI with positive control

## Data Availability

The datasets used and/or analyzed during the current study available from the corresponding author on reasonable request.
